# Hyaluronan and N-ERC/Mesothelin as Key Biomarkers in a Specific Two-Step Model to Predict Pleural Malignant Mesothelioma

**DOI:** 10.1371/journal.pone.0072030

**Published:** 2013-08-21

**Authors:** Filip Mundt, Gustav Nilsonne, Sertaç Arslan, Karola Csürös, Gunnar Hillerdal, Huseyin Yildirim, Muzaffer Metintas, Katalin Dobra, Anders Hjerpe

**Affiliations:** 1 Division of Pathology, Department of Laboratory Medicine, Karolinska Institutet, Stockholm, Sweden; 2 Stockholm Brain Institute and Osher Institute for Integrative Medicine, Department of Clinical Neuroscience, Karolinska Institutet, Stockholm, Sweden; 3 Stress Research Institute, Stockholm University, Stockholm, Sweden; 4 Eskisehir Osmangazi University Medical Faculty, Department of Chest Diseases, Eskisehir, Turkey; 5 Department of Lung Medicine, Thorax, Karolinska University Hospital, Stockholm, Sweden; University of Campinas, Brazil

## Abstract

**Purpose:**

Diagnosis of malignant mesothelioma is challenging. The first available diagnostic material is often an effusion and biochemical analysis of soluble markers may provide additional diagnostic information. This study aimed to establish a predictive model using biomarkers from pleural effusions, to allow early and accurate diagnosis.

**Patients and Methods:**

Effusions were collected prospectively from 190 consecutive patients at a regional referral centre. Hyaluronan, N-ERC/mesothelin, C-ERC/mesothelin, osteopontin, syndecan-1, syndecan-2, and thioredoxin were measured using ELISA and HPLC. A predictive model was generated and validated using a second prospective set of 375 effusions collected consecutively at a different referral centre.

**Results:**

Biochemical markers significantly associated with mesothelioma were hyaluronan (odds ratio, 95% CI: 8.82, 4.82–20.39), N-ERC/mesothelin (4.81, 3.19–7.93), CERC/mesothelin (3.58, 2.43–5.59) and syndecan-1 (1.34, 1.03–1.77). A two-step model using hyaluronan and N-ERC/mesothelin, and combining a threshold decision rule with logistic regression, yielded good discrimination with an area under the ROC curve of 0.99 (95% CI: 0.97–1.00) in the model generation dataset and 0.83 (0.74–0.91) in the validation dataset, respectively.

**Conclusions:**

A two-step model using hyaluronan and N-ERC/mesothelin predicts mesothelioma with high specificity. This method can be performed on the first available effusion and could be a useful adjunct to the morphological diagnosis of mesothelioma.

## Introduction

Malignant mesothelioma is an asbestos related cancer with a dismal prognosis, originating most commonly in the pleura or peritoneum. Diagnosis is based on morphological analysis of histological and cytological material. Metastatic adenocarcinoma or benign mesothelial hyperplasia are the main differential diagnoses, and ancillary techniques such as immunohistochemistry and electron microscopy are often required to reach a final diagnosis [1].

Initial symptoms of malignant mesothelioma are typically caused by a pleural effusion, and therapeutic thoracocentesis yields effusion material that can be analysed before biopsies are taken. In centres with access to cytologists experienced with mesothelioma diagnosis, effusion cytology in combination with immunocytochemistry, fluorescent in situ hybridization, and/or electron microscopy is sufficient for diagnosis in the majority of cases [1]–[4]. In those cases where these methods are not sufficient in order to reach a completely conclusive diagnosis, analyses of soluble biomarkers from effusions may be a useful complement to the morphological assessment.

Some biomarkers have previously been evaluated individually for mesothelioma diagnosis. Mesothelin related proteins (the ERC/mesothelin family), hyaluronan and osteopontin have been most frequently assessed, and some of them are also used in clinical practice.

ERC/mesothelin is a membrane bound 70 kDa precursor protein that can be cleaved to yield a 31 kDa peptide known as megakaryocyte potentiating factor (MPF or N-ERC/mesothelin) and a membrane bound ∼40 kDa protein (C-ERC/mesothelin). A transcript variant, arising from a frame shift, gives rise to an alternative C-terminus in an excreted soluble mesothelin-related protein [5], [6]. In this article we will refer to megakaryocyte potentiating factor as *N-ERC/mesothelin*, while reserving the term *C-ERC/mesothelin* for fragments detected by the MESOMARK™ ELISA kit. The mesothelin family shows specificity for mesothelioma in effusions, serum and plasma [7]–[10]. Hyaluronan is a linear polysaccharide associated with mesothelioma with high specificity but limited sensitivity [11]–[14]. Increased levels of osteopontin have also been linked to mesothelioma [15]–[18], but also several other conditions [19], resulting in low diagnostic specificity.

In addition to these established biomarkers, we aimed to investigate three putative markers: thioredoxin, syndecan-1, and syndecan-2. The redox enzyme thioredoxin has been found to be upregulated in mesothelioma cell lines and biopsies, as detected by subtractive hybridisation, microarray and immunohistochemistry [20]–[23]. Syndecan-2 is a membrane bound proteoglycan associated with mesenchymal tissues [24] while syndecan-1, in contrast, is a marker for adenocarcinoma as compared to mesothelioma [24], [25].

Individual mesothelioma biomarkers lack either sensitivity or specificity and there is a need for new markers or combinations of markers to aid morphological classification. This study aimed to build and validate a predictive model for mesothelioma diagnosis, using the detection of soluble biomarkers in pleural effusions.

## Materials and Methods

### Ethics Statement

All patients included had signed a written informed consent and the study was approved by the regional ethical committees of Stockholm and Eskisehir Osmangazi University.

### Study Participants in the Model Generation Dataset

**Figure 1:**
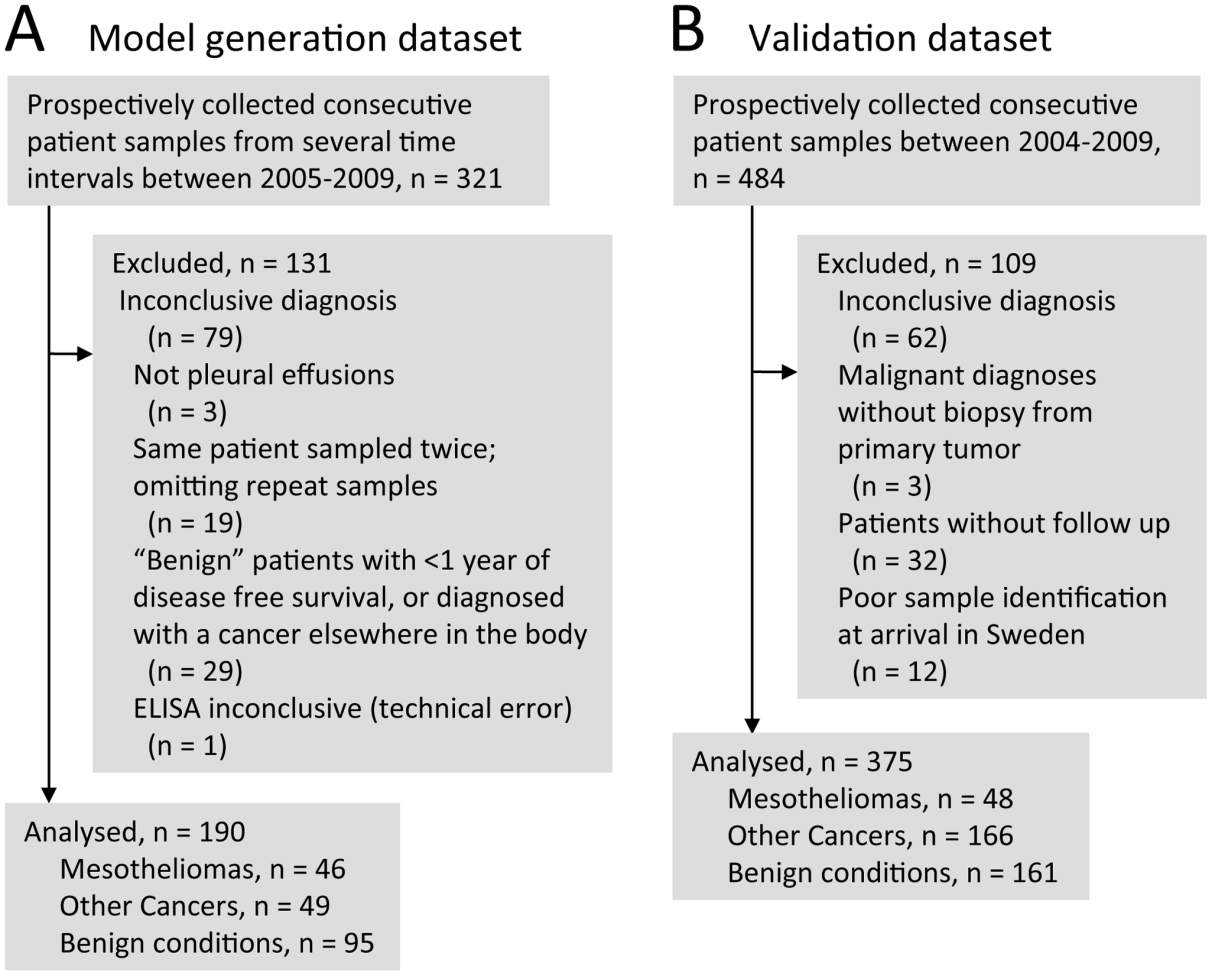
Exclusion and inclusion criteria for the studied populations. Flow chart showing patient exclusion and inclusion for the **A)** model generation dataset and **B)** the validation dataset.

For model generation, we analysed effusions received at the Department of Pathology and Cytology, Karolinska University Hospital in Huddinge, Sweden – a tertiary referral centre. The material was collected as consecutive samples in different time periods between 2005 and 2009 and consisted of pleural effusions from 321 patients. Cell free supernatants were kept at −20°C without additives. All mesothelioma diagnoses were established by histopathology, using comprehensive immunohistochemistry [26], and/or electron microscopy, the latter performed on biopsies or effusion cell pellets. Pleural involvement of the metastatic tumours was established by histopathology and/or cytology, using immunohistochemical panels. Hyaluronan had in some cases been used as an adjunctive diagnostic marker for mesothelioma, but the diagnoses were established in all cases using the above-mentioned independent methods. None of the non-mesothelioma patients were diagnosed with a mesothelioma during the subsequent three years and none of the mesothelioma samples represented disease recurrence. Patients with malignancies other than mesothelioma included mainly metastases from primary lung and breast adenocarcinoma (table 1). A group of benign pleural fluids, including mostly exudates, but also some transudates, was collected consecutively. Further information on the etiology of these fluids was not available, but all patients were still alive one year after the collection of fluids and had not, in this time, been registered in the pathology database as having a diagnosis of cancer (figure 1A).

**Table 1:** Demographic data of the analysed datasets.

*Model generation dataset*	Number of patients	Male (%)	Female (%)	Age, median (IQR)
Benign	95	65 (68)	30 (32)	69 (54–80)
Other cancer	49	15 (31)	34 (69)	68 (62–77)
Lung cancer	16			
Cancer of unknown primary	10			
Breast cancer	8			
Ovarial & Fallopian tube cancer	5			
Gastric cancer	5			
Pancreatic cancer	3			
Other malignancies	2			
Mesothelioma	46	41 (89)	5 (11)	70 (62–80)
***Validation dataset***	**Number of patients**	**Male (%)**	**Female (%)**	**Age, median (IQR)**
Benign	161	113 (70)	48 (30)	59 (48–71)
Other cancer	166	96 (58)	70 (42)	64 (56–72)
Lung cancer	85			
Cancer of unknown primary	11			
Breast cancer	23			
Ovarian cancer	8			
Gastric cancer	6			
Prostate cancer	3			
Bladder cancer	3			
Other malignancies	27			
Mesothelioma	48	17 (35)	31 (65)	64 (54–70)

Age (IQR = interquartile range) and patient sub-grouping in the model generation data set and validation dataset. The high proportion of female mesothelioma patients in the validation dataset is most likely due to environmental asbestos and erionite exposure, which is related to geographical distribution and is in concordance with a previous study [54].

### Study Participants in the Validation Dataset

A second study, for external validation, consisted of material collected prospectively and consecutively at the Medical Faculty of Eskisehir, Department of Chest Diseases, Turkey – also a tertiary referral centre. A total of 484 effusions were frozen and stored at −80°C without additives between 2004 and 2009. The majority of patients with cancer other than mesothelioma were diagnosed as metastases from either lung or breast adenocarcinoma (table 1). Benign diagnoses were mainly tuberculosis, pleuritis or transudate due to congestive heart failure. All mesothelioma were diagnosed with histopathology, supported by a limited immunohistochemistry panel. All cases with metastatic involvement of the pleura had diagnoses based on histological material; however, the pleural involvement was sometimes also shown by cytological assessment. Benign diagnoses were mainly clinical, and some cases lacked long-term follow-up (figure 1B).

### Enzyme-Linked Immunosorbent Assays (ELISA)

ELISA kits for human N-ERC/mesothelin (code no. 99666/7–16 assay), osteopontin (code no. 27158) and thioredoxin (code no. 27417) were all purchased from Immuno-Biological Laboratories Co., Ltd., Japan. The ELISA kit for C-ERC/mesothelin was purchased from FDI Fujirebio Diagnostics, Inc. (MESOMARK™). The ELISA kit for hyaluronan was from Corgenix (ref. no. 029–001) and that for syndecan-1 from Diaclone (cat. no. 950.640.096). Analyses were performed according to the manufacturers’ instructions using a precision TM XS Microplate Sample Processor from Bio-Tek Instruments Inc., Vermont, USA. The syndecan-2 ELISA was performed as described earlier [27] with the exception that we used 5 µg of the monoclonal mouse anti-human syndecan-2 antibody (10H4) [28] for coating. The effusions were diluted to 1∶2000 (hyaluronan), 1∶200 (N-ERC/mesothelin), 1∶200 (C-ERC/mesothelin), 1∶500 (osteopontin), 1∶2 (syndecan-2), 1∶3 (syndecan-1) or 1∶3 (thioredoxin) in buffers used as reagent blanks, according to pre-optimization experiments (data not shown). All samples were analysed in duplicate at one laboratory (Karolinska Institutet) by investigators blinded to the final diagnoses.

### High-Performance Liquid Chromatography (HPLC)

To ensure correct measurements in a wide range, all elevated values of hyaluronan (>25 µg uronic acid/mL) were verified by HPLC, using a protocol developed by us in 1986 [29]. Peaks at 231 nm were compared to an external standard and recorded as µg hyaluronan-derived uronic acid/mL (µg UA/mL) (approx. 0.3 times the total theoretical weight of water-free hyaluronan).

### Western Blot

Since the epitopes targeted by the osteopontin ELISA bridge a thrombin cleavage site, we investigated whether effusion osteopontin was present as the full length protein or in a cleaved form. Six mesothelioma samples with levels of osteopontin undetectable by ELISA, but high levels of both hyaluronan and N-ERC/mesothelin and one sample with a high osteopontin level (∼17 µg/mL) were analysed by western blot. Effusions were prepared following the Invitrogen™ protocol for NuPAGE® Novex® Bis-Tris Mini Gels and used BioRad® pre-cast gels (4–15%). Briefly, equal volumes of samples were denatured at 95°C for 5 minutes and separated for 1.5 h (100 V) in MOPS buffer (Invitrogen®). Transfer to a PVDF membrane was performed overnight at 30 V. Non-specific binding was blocked using 5% skimmed milk buffer and the membrane was blotted with a primary polyclonal rabbit antibody against human osteopontin (1∶800, 0.05% TBST) (kindly provided by Dr. Barbro Ek-Rylander) followed by an alkaline phosphatase conjugated goat anti rabbit polyclonal IgG_1_ antibody (1∶800, 0.05% TBST) (A8025, Sigma-Aldrich®). The membrane was washed in 0.05% TBST and visualised with NBT/BCIP.

### Retest-reliability of the Biomarkers

When decoding the patient samples of the model generation dataset, 19 patients appeared twice. While only including the measurements from the first obtained effusion for model generation, these paired samples were used to assess retest-reliability of the biomarkers using Spearman’s rank correlation test.

### Model Generation and Validation

Log-transformation was used since it yielded more normal distributions of all biomarkers (data not shown). Values of 0 were assigned the value 0.1 at log-transformation. Logistic regression was used to determine univariate odds ratios for each biomarker with mesothelioma as the dependent variable (coded as 1 or 0) and each biomarker as the independent variable. Cut-offs to simultaneously optimise sensitivity and specificity were determined as well as positive likelihood ratios.

Variable inclusion in a multivariate predictive model was determined by a stepwise forward selection procedure implemented in 1000 bootstrap iterations. The bootstrap selection procedure counteracts overfitting on the model generation dataset by performing variable selection in new datasets (iterations) generated randomly from the existing one. Discrimination is illustrated by ROC curves. Calibration was analysed using calibration plots comparing predicted and observed probabilities and by Spiegelhalter’s z score [30] then further characterised by Nagelkerke’s R^2^ and Brier scores. We compared and selected models based on the net reclassification improvement which compares model classification, and the integrated discrimination improvement, which is a measure of the difference in predicted probabilities between true positive and true negative cases. Integrated discrimination indexes were calculated as the difference between discrimination slopes [31], [32].

The statistics for our present manuscript was partly obtained from a statistical company (Statisticon Inc., www.statisticon.se). Analyses were performed using R-software (v. 2.12.2) [33] with the packages boot.StepAIC [34] OptimalCutpoints [35], rms [36] and GraphPad Prism (GraphPad Software, Inc., v. 5.04).

## Results

### Biomarker Expression in the Model Generation Dataset

**Figure 2:**
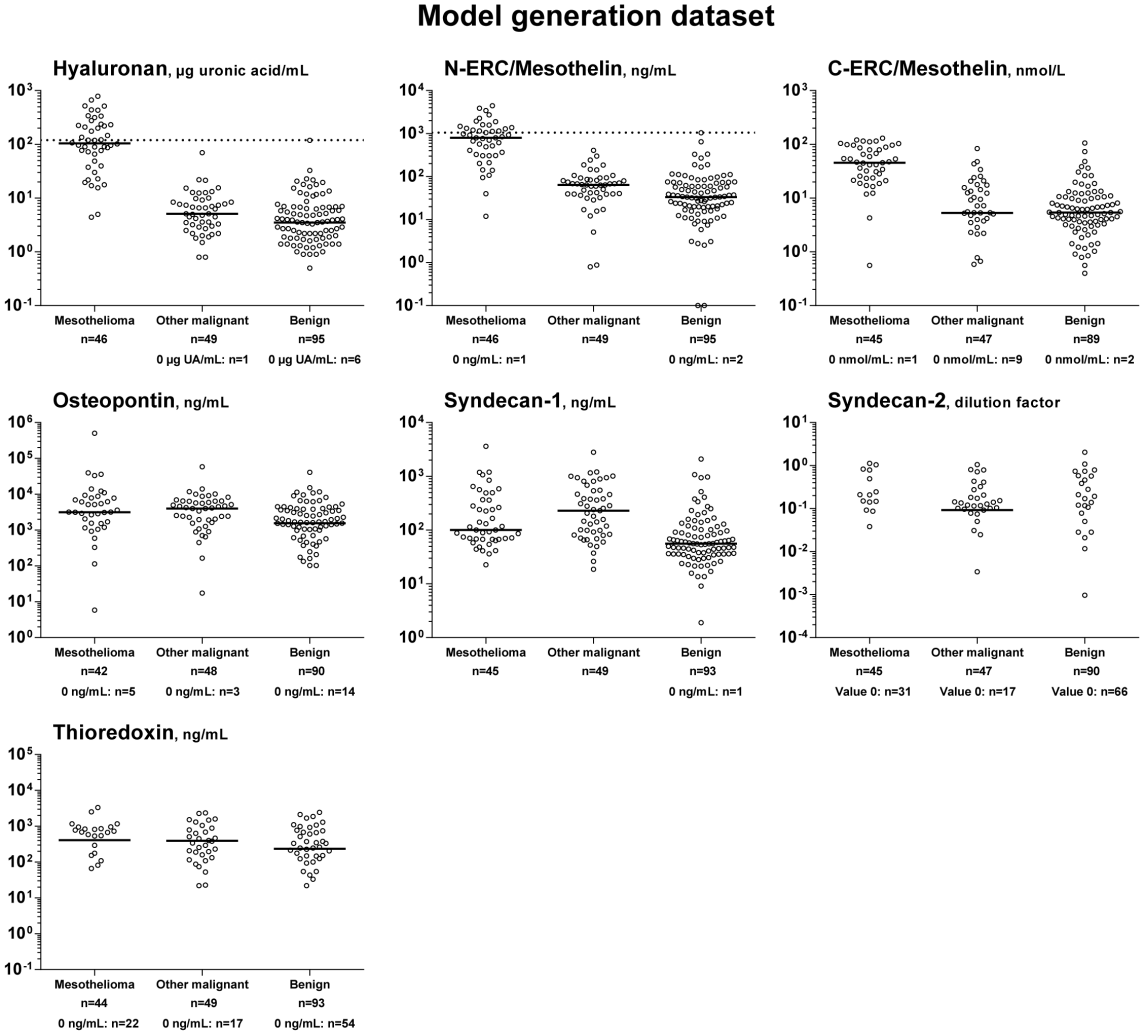
Biomarker expressions in diagnostic sub-groups in the model generation dataset. Levels of hyaluronan, N-ERC/mesothelin, C-ERC/mesothelin, osteopontin, syndecan-2, syndecan-1, and thioredoxin in patients with malignant pleural mesothelioma, other malignant pleural disease, or benign effusions. Values of 0 (i.e. below the detection limit) are tabulated for each group under the respective graph, as they cannot be shown on a logarithmic scale. Dotted line represents cut-off values. Horizontal lines represent medians. N_tot_/biomarker<190 indicates the exclusion of patients from individual analyses due to insufficient material (e.g. thioredoxin, n = 186).

Seven biomarkers were tested in effusions from 190 patients (figure 2). Levels of hyaluronan, osteopontin, N-ERC/mesothelin and C-ERC/mesothelin were comparable to those reported earlier, supporting the validity of our results [7], [9], [11], [16], [37]–[44]. Values obtained for the different groups were unaffected by storage time, indicating that epitopes were stable and well preserved. Specific western blots showed that osteopontin was not degraded prior to ELISA analysis (data not shown).

**Figure 3:**
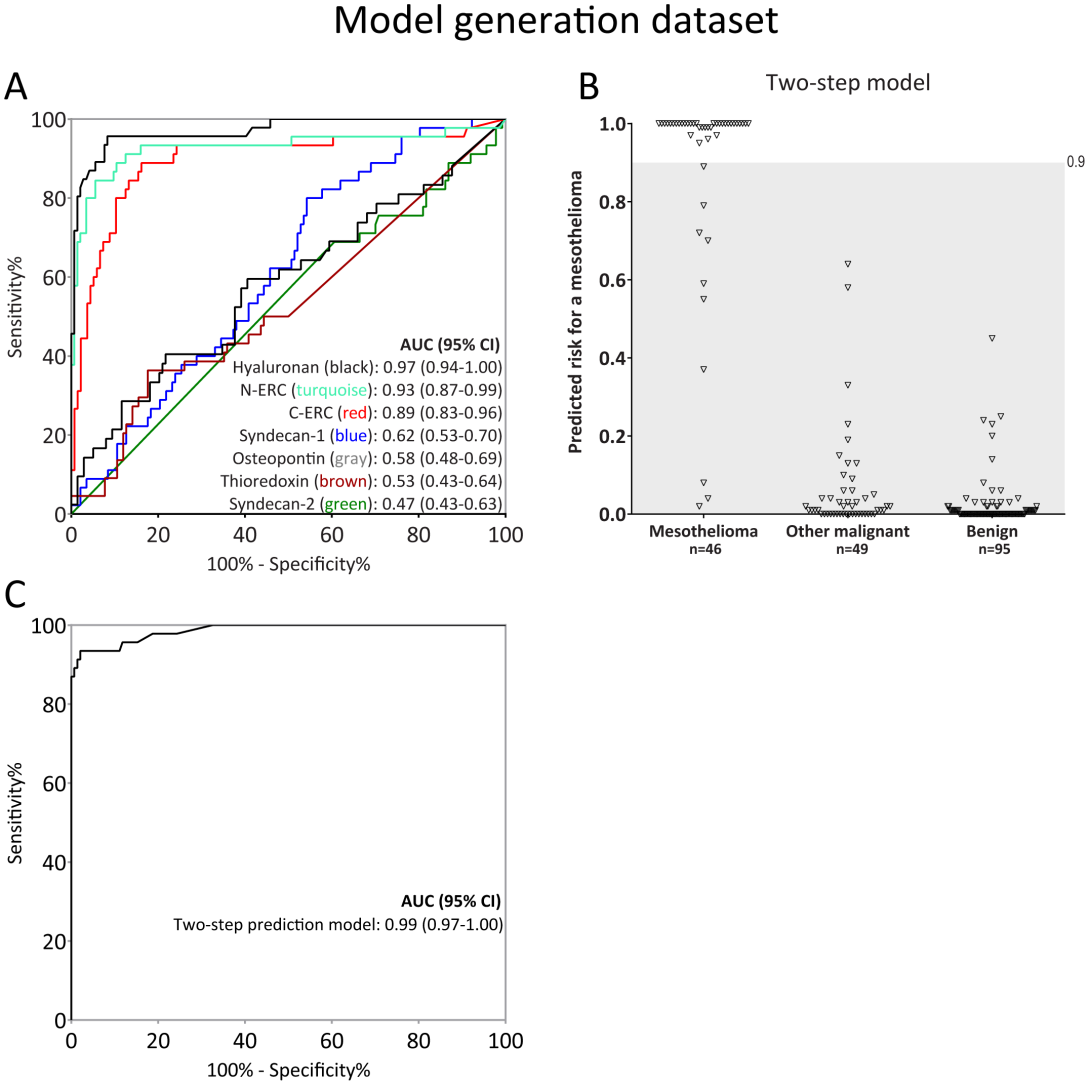
Model generation and performance. **A)** ROC curves showing sensitivity and specificity for individual biomarkers. **B)** Predicted risk values from two-step model based on hyaluronan and N-ERC/mesothelin. Cases with a predicted risk>0.9 were considered positive (above shaded area). **C)** ROC curve for the two-step model.

The chemical markers most strongly predictive of mesothelioma were hyaluronan, N-ERC/mesothelin, and C-ERC/mesothelin. Syndecan-1 also showed a significant odds ratio, although with lower magnitude. Syndecan-2, osteopontin and thioredoxin were not significant (table 2 and figure 3A).

**Table 2:** Odds ratios for biomarkers in the model generation dataset.

*Biomarker*	*Odds ratio (95% CI)*	*p* * (Odds ratio ≠ 1)	*Highest sensitivity/specificity*	*Likelihood ratio positive (95% CI)*
Log Hyaluronan	8.82 (4.82–20.39)	<0.0001	0.93/0.92	12.24 (6.90–21.70)
Log N-ERC/mesothelin	4.81 (3.19–7.93)	<0.0001	0.89/0.90	8.53 (5.23–13.93)
Log C-ERC/mesothelin	3.58 (2.43–5.59)	<0.0001	0.87/0.85	5.61 (3.73–8.45)
Log Syndecan-1	1.34 (1.03–1.77)	0.03	0.58/0.56	1.30 (0.95–1.78)
Log Syndecan-2	1.32 (0.96–1.94)	0.12	0.76/0.37	1.20 (0.98–1.49)
Log Osteopontin	1.06 (0.94–1.22)	0.38	0.60/0.59	1.47 (1.06–2.02)
Log Thioredoxin	1.03 (0.92–1.15)	0.61	0.50/0.56	1.13 (0.80–1.29)

Association of biomarkers with malignant mesothelioma and diagnostic performance characteristics. Interquartile odds ratios from logistic regression models are shown with associated p-values. Sensitivity and specificity are shown at a point which optimises both measurements, and the associated positive likelihood ratio is also shown. Odds ratios were calculated using log_10_ transformed values.

* Based on a one-tailed statistical analysis.

Retest reliability in patients tested twice was high for N-ERC/mesothelin, hyaluronan, Syndecan-1 and C-ERC/mesothelin (R^2^ of 0.97, 0.86, 0.84 and 0.79, respectively) while thioredoxin showed moderate reliability (R^2^ of 0.39) and osteopontin low (R^2^ of 0.01) (figure S1 in file S1).

### Model Development

Variables for inclusion in a multivariate predictive model were selected in 1000 bootstrap samples, depending on their ability to classify a malignant mesothelioma. Hyaluronan and N-ECR/mesothelin were selected in all iterations, whereas none of the other biomarkers were selected even once. Hyaluronan and N-ERC/mesothelin were thus chosen for inclusion in a logistic model.

Testing for co-linearity revealed a moderate coefficient of determination of 0.35 (p<0.0001) between hyaluronan and N-ERC/mesothelin (figure S2 in file S1). Cut-off values at 100% specificity were applied as a first step, for each biomarker, in order to avoid linear separation in the logistic model. Without these cut-off values, hyaluronan separated the mesothelioma patients to such an extent that no other biomarker could contribute significantly, thus preventing a combinatorial approach. The following cut-off levels were used: 120 µg UA/mL for hyaluronan and 1050 ng/mL for N-ERC/mesothelin (figure 2). In essence, we established a two-step model combining N-ERC/mesothelin and hyaluronan on the patients expressing moderate levels of selected biomarkers. This yielded a b_0_ (correction coefficient) of −12.42 and b_i(HAlog)_ of 4.71 and b_i(N-ERClog)_ of 2.71 in the logistic model (*p*(1−x) = (*e* ^(b0−Σxi*bi)^)/(1+*e* ^(b0−Σxi*bi)^)). The generated predicted risks of a mesothelioma in the present data set (p-values) are plotted in figure 3B and the corresponding ROC-curve is shown in figure 3C.

Sensitivity at 100% specificity of hyaluronan, N-ERC/mesothelin and the two-step model were 45.7%, 37.9% and 87.0%, respectively. The categorical net reclassification improvement increased by 29% (95% CI: 14–44%) and 40% (95% CI: 22–58%) and the integrated discrimination index increased by 12% (95% CI: 7–17%) and 20% (95% CI: 13–28%) when using the two-step model compared to only hyaluronan or N-ERC/mesothelin respectively, reflecting greater diagnostic performance.

### Model Validation

**Figure 4:**
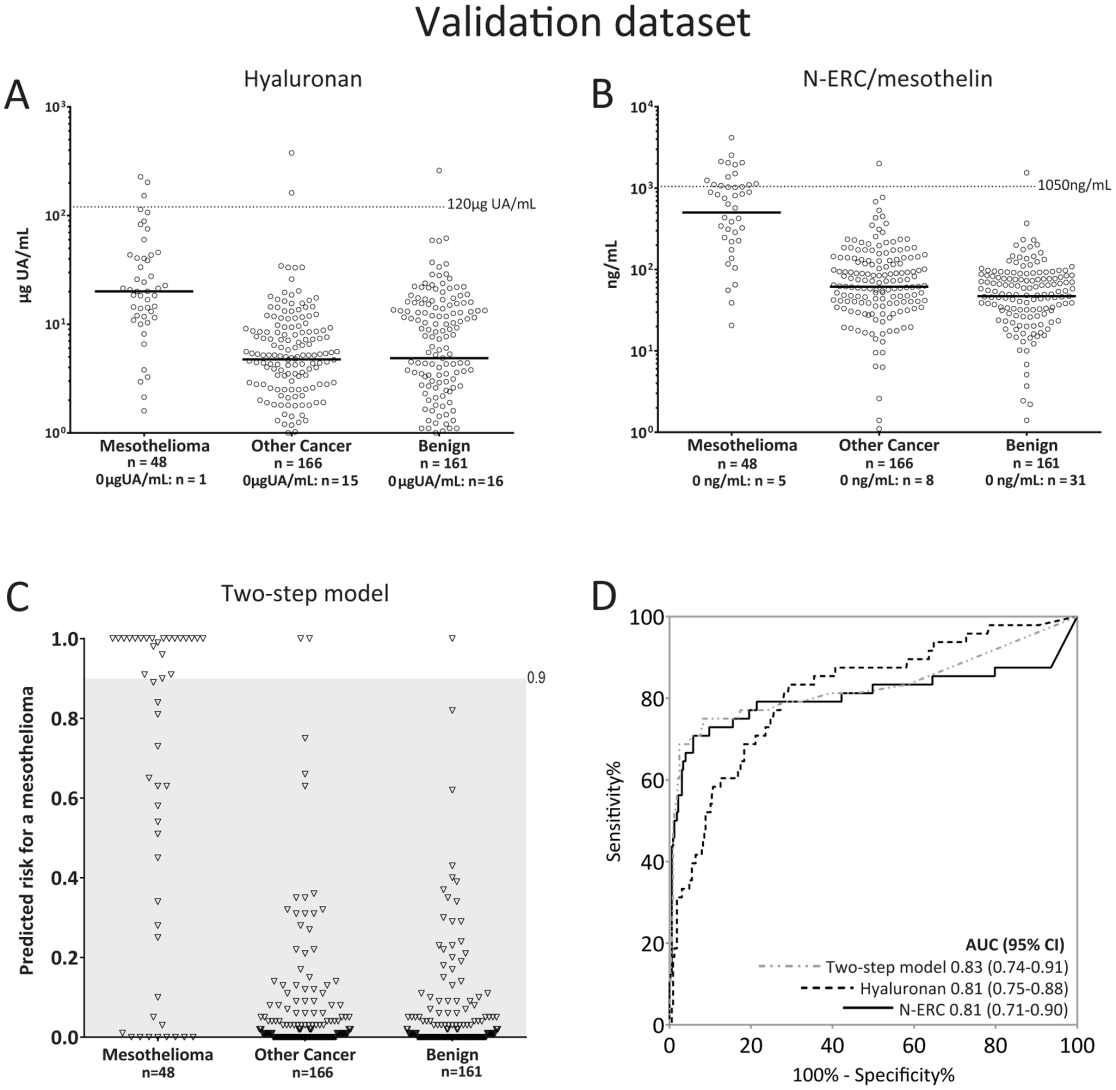
Validation of hyaluronan and N-ERC/mesothelin levels as well as the two-step model. **A)** and **B)** Levels of hyaluronan and N-ERC/mesothelin, respectively, in the validation dataset. The dotted line represents cut-off values. **C)** Predicted risk values from the two-step predictive model. Cases with predicted risks>0.9 were considered positive (above shaded area). **D)** ROC curves generated from N-ERC/mesothelin (solid black line) and hyaluronan (dotted black line) as single markers or combined in the two-step model (dotted grey line).

We proceeded to analyse hyaluronan and N-ERC/mesothelin and test the two-step model in a validation dataset consisting of 375 effusions (figure 4A and B); corresponding ROC-curves are shown in figure 4D.

In the validation dataset, the two-step model had a sensitivity and specificity (at a predicted risk>0.81) of 52.0% and 98.7% respectively (figure 4C). Sensitivities of hyaluronan and N-ERC/mesothelin at the same high specificity (98.7%) were 16.0% and 44.0%, respectively. The two-step model had better classification accuracy and the net reclassification index showed it to be superior to N-ERC/mesothelin and hyaluronan as individual markers (table S1 in file S1). The categorical net reclassification improvement increased by 31% (95% CI: 15–47) and 14% (0.7–28) when comparing the two-step model against hyaluronan or N-ERC/mesothelin, respectively. Additionally, the continuous net reclassification improvement increased by 83% (95% CI: 111–56) and 40% (14–65) when comparing the two-step model against hyaluronan or N-ERC/mesothelin, respectively. Furthermore, the integrated discrimination index increased using the two-step model by 25% (95% CI: 17–34) compared to hyaluronan, while it only increased by 5% (95% CI: −2–12) compared to N-ERC/mesothelin.

**Figure 5:**
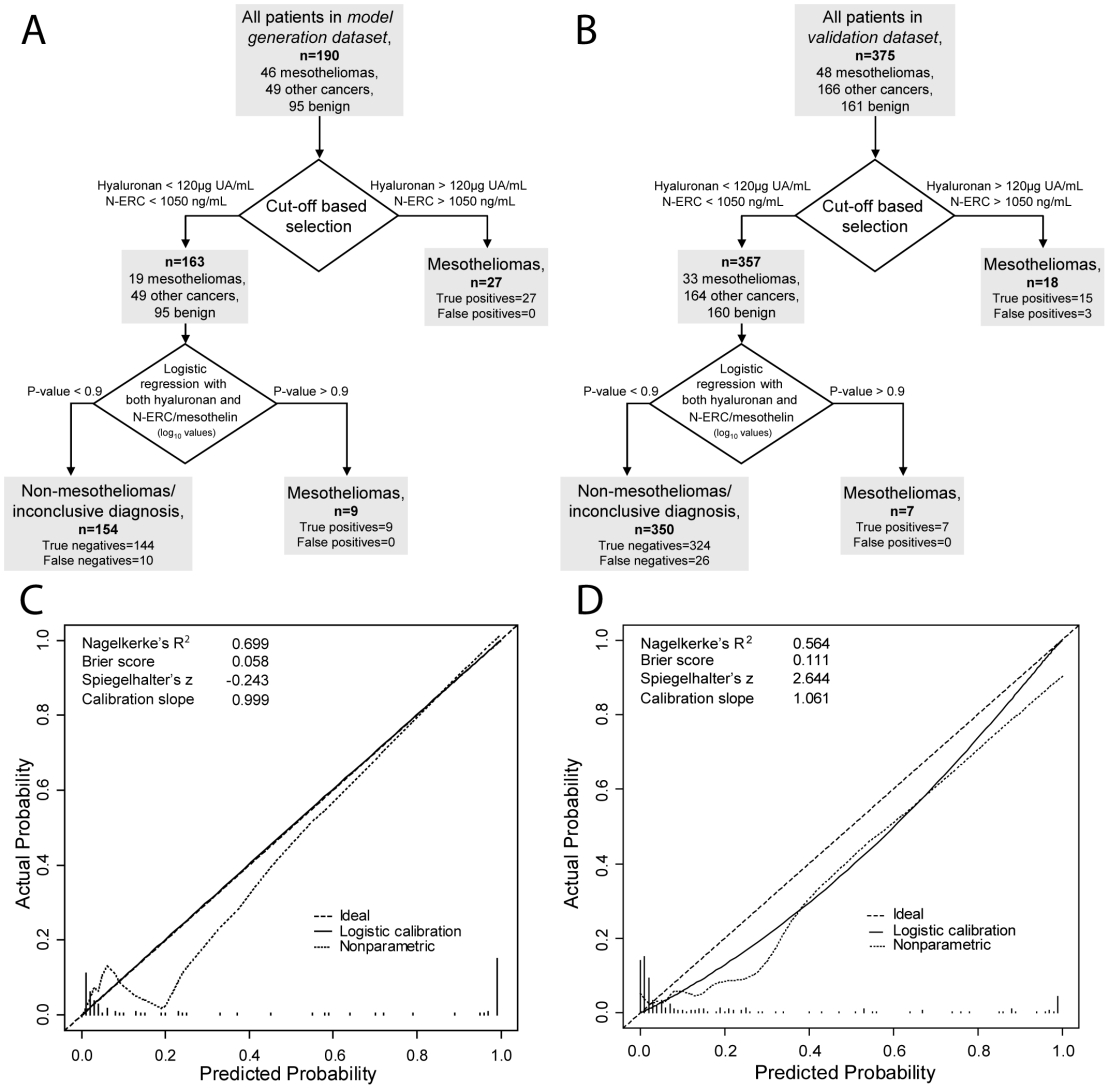
Schematic representation and model calibration of both datasets. **A)** Schematic presentation of the two-step model and its performance on the model generation dataset. **B)** Schematic presentation of the two-step model and its performance on the validation dataset. In both **A)** and **B)**, after the logistic regression a predicted risk value>0.9 indicates additional mesothelioma cases compared to hyaluronan or N-ERC/mesothelin alone. **C)** and **D)** Calibration plots showing the agreement between observed outcomes (y-axis) and predictions (x-axis) in the model generation dataset and validation dataset respectively.

**Figure 6:**
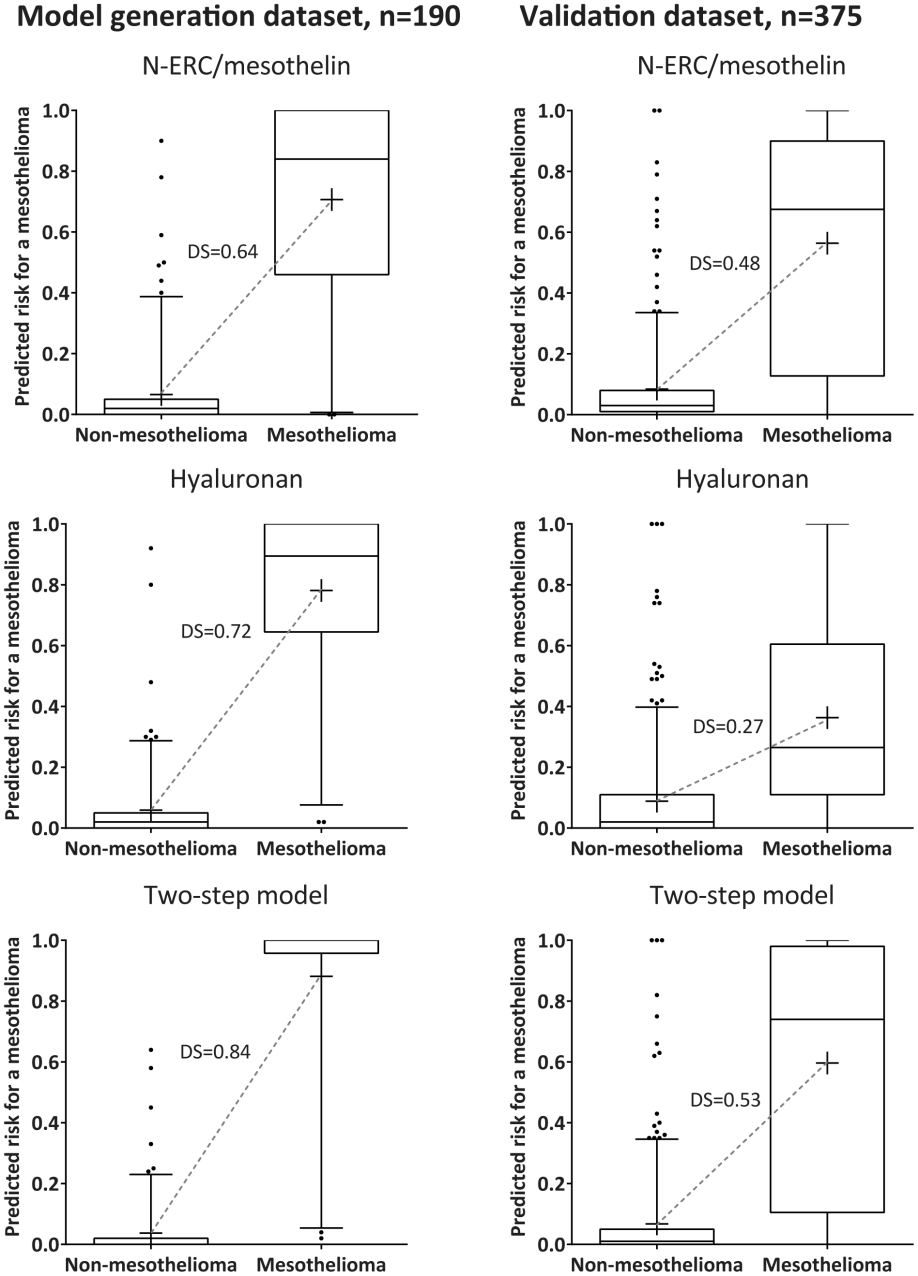
Discrimination slopes between mesothelioma and non-mesothelioma for the different predictors in both datasets. Box plots of predicted probabilities using hyaluronan, N-ERC/mesothelin or the two-step model on the model generation dataset and validation dataset. The mean is denoted by a “+”, whiskers indicate the 5^th^ and 95^th^ percentiles, while dots are outliers. Grey dotted lines represent the discrimination slope (DS) which is equivalent to the integrated discrimination index. The differences in the discrimination slopes correspond to the integrated discrimination index [31], [32]. As an example, the highest possible discrimination slope would be 1 (100%) and comparing N-ERC/mesothelin with the two-step model in the model generation dataset shows that the integrated discrimination index increases with 20% when applying the two-step model (DS_Model_ 0.84 minus DS_N-ERC_ 0.64).

Schematic flow charts of the two-step model on respective datasets are shown in figure 5A and B. The model retained good calibration in the validation dataset, although with some shrinkage compared to the model generation dataset (figure 5C and D). Discrimination slopes, which are equivalent to calculated integrated discrimination indexes, are plotted per dataset for hyaluronan, N-ERC/mesothelin and the two-step model in figure 6.

In the validation dataset the two-step model indicated a predicted risk of >0.75 in three cases diagnosed as metastatic cancer and in two cases considered to be benign, raising a suspicion that they could in fact correspond to malignant mesothelioma. Paraffin embedded tumour tissue was available to allow extendedimmunohistochemical analysis (15 markers) in two of the cases originally diagnosed as carcinoma. This analysis indicated that they both were indeed mesothelioma. In the last case there was a history of malignant disease but with no histological material available from the pleural tumour (table S2 in file S1). The two benign samples with high predicted risk values had no long term follow-up, and it may well be that all these five cases with high predicted risk values were indeed occult mesothelioma.

Paraffin blocks were also available in seven of the twelve cases in the validation mesothelioma group where the model indicated low predicted risk of a mesothelioma (predicted risk<0.1). The above-mentioned immunohistochemical panel indicated that one of them was an adenocarcinoma; the remaining tumours reacted like mesothelioma (table S2 in file S1).

## Discussion

Early diagnosis of malignant mesothelioma is crucial for the choice of treatment and may influence patient survival. Reaching a definitive diagnosis of mesothelioma is often a time consuming task, delaying the clinical management of the patient by several months; for example a pleural biopsy was among the Swedish mesothelioma cases obtained up to 11 months after the initial effusion.

Ancillary methods allow a more accurate diagnosis as evidenced by the widespread use of and molecular analyses in many cancers. Effusion biomarkers have the additional advantage of being available for analysis at the time of the first clinical manifestation of symptoms for most patients. The most studied soluble mesothelioma marker to date is the C-ERC/mesothelin fragment: in pleural effusions the AUC span from 0.72 to 0.88 in various studies [39]–[43], however, most studies lack external validation.

In this study, we have investigated four established and three putative biomarkers; our main finding was that the combination of N-ERC/mesothelin and hyaluronan in a two-step model gives better prediction than either marker on its own as shown by increasing net reclassification improvement and integrated discrimination index. This finding was confirmed by external validation in a second patient dataset. Judging from the odds ratios and ROC curves, N-ERC/mesothelin was a better predictor compared to C-ERC/mesothelin; a finding validated with bootstrap selection. The difference between the two mesothelin fragments is marginal; a finding that is in agreement with studies performed on blood [45]–[47]. To our knowledge we are first to evaluate N-ERC/mesothelin in pleural effusions.

The measured concentrations of both mesothelin fragments have been shown to correlate to the amount of tumour tissue [48], i.e., higher values being obtained in more advanced stages. Furthermore, while hyaluronan is produced by all phenotypes of mesothelioma [11], elevated mesothelin levels are mostly seen in epithelioid and mixed phenotypes [46]. The two materials studied were, however, both consecutive and their clinical stages reflected the entire panorama when presenting with the first effusion. Furthermore, the ROC curves obtained, when applying the two-step model on the two data sets, showed only marginally improved AUCs. When, however, the diagnostically important region of high specificity is considered, the net reclassification improvement and the integrated discriminatory index clearly demonstrate the improved performance of the model compared to the individual biomarkers.

In the validation dataset, the two-step model showed less discrimination than in the model generation dataset (figure 6). High specificity was however retained at validation, and that is likely to be more important in the clinical use of a predictive model for mesothelioma. It should also be noted that the relative contribution of N-ERC/mesothelin in the two-step model was greater in the model validation dataset than in the model generation dataset. One possible contributing factor is that hyaluronan was used in some cases to aid diagnosis in the model generation dataset, although all diagnoses were established by independent methods. Difference in tumour stages between the two populations could also be part of the explanation. Unfortunately we lack this information for most cases. The diagnostic accuracy was ensured in the model generation dataset by histopathology, with comprehensive immunohistochemical analyses and/or electron microscopy. All cases with morphologically unconfirmed diagnoses were excluded. The diagnostic reference for the validation dataset was less stringent. All mesothelioma cases were verified by histopathology but with more limited immunohistochemical support. We re-evaluated a set of patients where there was a discrepancy between our proposed model and initial clinical diagnosis. When re-evaluating these patients with an extended immune-panel the initial diagnosis was rightfully challenged in a few cases (table S2 in file S1). However, since we only had the possibility to apply the extended immune-panel on a sub-set of patients (we do not have the histological material at our disposal for all patients), the conversion of only a few diagnoses would infer a bias and is therefore something we would prefer to refrain from doing. Additionally, we would like to suggest that the difference in model performance may be partly due to model shrinkage, i.e. a choice of parameters that was optimal in the generation dataset but less optimal in the validation dataset.

In three cases of benign asbestos pleuritis a mesothelioma was diagnosed several months later. In two of these cases the two-step model gave moderately raised predicted risks of a mesothelioma of 0.89 and 0.58. They may therefore represent an early stage of the tumour, indicating that these biomarkers are detectable already in early stages of the disease. We however lack information about tumour stage and grade in most cases, which limits our understanding of these biomarkers possibility to detect early mesothelioma cases.

The combined analysis of hyaluronan and C-ERC/mesothelin in pleural effusions has previously been reported in a small set of patients [9], concluding that there was little or no interest in combining the two. This is similar to experiences with serum analyses, as presented in a recent review [49]. However in the present study, increased predictive performance was achieved when using N-ERC/mesothelin instead of C-ERC/mesothelin together with cut-off based selection in order to avoid linear separation.

Osteopontin had poor predictive ability and low retest-reliability which calls into question the reliability of this analyte and the validity of the ELISA. However, Western blotting identified osteopontin to be mostly in its uncleaved form and the levels are comparable to those described in previous studies [16], [44]. Osteopontin occurs in isoforms [50], and we cannot exclude the possibility that better predictability might be seen with a different ELISA [51]. In this study the amount of soluble syndecan-2, was generally low, with a large proportion of zero values, whereas syndecan-1 was present at measurable levels in most effusions. This difference may relate to the cellular distribution of syndecan. In epithelioid mesothelioma cells, syndecan-2 is mainly found in the cytoplasm, while syndecan-1 is mainly located at the cell membrane [25], from where it may be shed into extracellular fluids. Recently, thioredoxin concentrations in serum were shown to be predictive of mesothelioma [52]. In the present study, thioredoxin in effusions were not predictive of mesothelioma, suggesting that the turnover of thioredoxin may differ between effusion fluids and serum.

We propose a two-step model based on N-ERC/mesothelin and hyaluronan. This model has the practical benefit that it is possible to screen suitable cases with just one of the two markers, while the second marker is only needed to evaluate the sub-set of samples unresolved by the first. To clarify, if the first compound (hyaluronan or N-ERC/mesothelin) shows a value above the predetermined cut-off, then it will support a mesothelioma diagnosis. If it does not, then the sample can be analysed with the second biomarker, again applying an upper cut-off supporting the diagnosis of a mesothelioma. If the patients is below both cut-offs, prediction will rest on the logistic model using information from both markers (Appendix in file S2). This approach extended the number of correctly classified mesothelioma patients by 9 in the model generation dataset and by 7 in the validation dataset (figure 5A and B).

The proposed two-step model supports the diagnosis of a malignant mesothelioma based on pleural effusions and can be integrated into the clinical workup, together with routine morphology, immunocytochemistry and/or electron microscopy of cell pellets. This allows, in most cases, a definite diagnosis based on the first effusion, the diagnosis being sufficient to guide the choice of chemotherapy. When surgery is considered, biopsy sampling may be necessary to evaluate a possible sarcomatoid tumour component, but this will only apply in a small proportion of patients.

The diagnosis can in this way be obtained even months before a biopsy is considered. Still it seems as if the mere presentation with an effusion often reflects an already advanced stage of the disease. Even earlier detection would necessitate presymptomatic analyses of blood samples [53]. The utility of this model as an adjunct to effusion cytology may be further improved by additional biochemical markers. A search for such novel biomarkers, using proteomics screening of pleural effusions, is on-going in our laboratory.

## Supporting Information

File S1 **Includes table S1 and S2 as well as figure S1 and S2.**
(DOC)

File S2 **Appendix with practical examples of how to use the two-step model.**
(DOCX)

Supporting Information S1 **All raw data acquired from the ELISA analyses on both materials can be downloaded from the Dryad online repository (DOI:** http://dx.doi.org/10.5061/dryad.h122b **).**
(DOC)
