# Supplementary material for: Hyaluronan and N-ERC/Mesothelin as Key Biomarkers in a Specific Two-Step Model to Predict Pleural Malignant Mesothelioma
Source: PLoS One. 2013 Aug 21;8(8):e72030. doi: 10.1371/journal.pone.0072030 (PMC3749097; doi:10.1371/journal.pone.0072030)

**Supplementary material**

This file has been provided by Mundt F, et al. to give supplementary information about their study:

*Pleural effusion biomarkers for diagnosis of malignant mesothelioma*

Supplement to: Mundt F. et al., A biomarker panel predicting mesothelioma

**Index**

Table: S1 & S2 page 3 & 4

Figures: S1 & S2 page 5 & 6

***Table S1*** Contingency table of the two-step model, N-ERC/mesothelin and hyaluronan. Sensitivity is reported after adjustment for high specificity (99%). The increase in net reclassification (NRI) is specified when using the two-step model over single biomarkers on the validation dataset.

| ***Cut-off (predicted risk)*** | ***Two-step model*** | | *total* | ***N-ERC/mesothelin*** | | *total* | ***Hyaluronan*** | | *total* |
| --- | --- | --- | --- | --- | --- | --- | --- | --- | --- |
|  |  | Non-mesothelioma |  |  | Non-mesothelioma |  |  | Non-mesothelioma |  |
|  | Mesothelioma |  |  | Mesothelioma |  |  | Mesothelioma |  |  |
| Positive | 25 | 4 | *25* | 21 | 2 | 23 | 8 | 3 | *11* |
| Negative | 23 | 323 | *350* | 27 | 327 | 360 | 40 | 324 | *364* |
| *total* | *48* | *327* |  | *48* | *327* |  | *48* | *327* |  |
| Sensitivity | 52.1% | |  | 43.8% | |  | 16.7% | |  |
| Specificity | 98.7% | |  | 99.4% | |  | 99.1% | |  |
| Net reclassification (categorical; 95% CI) | 14% (0.7-28) | | | | | | | | |
|  | 31% (15-47) | | | | | | | | |
|  |  |  |  |  |  |  |  |  |  |
| Net reclassification (continuous; 95% CI) | 40% (14-56) | | | | | | | | |
|  | 83% (111-56) | | | | | | | | |
| *(Between two-step model&N-ERC or HA)* |  | | | | | | | | |

***Table S2 An extended immunohistochemistry panel***

Parameters in favour of adenocarcinomas (shaded grey): BerEp4, monoclonal CEA, CD15, Sialyl-TN, TTF1. Parameters in favour of mesothelioma: EMA in cell membrane, Calretinin, HBME1, Thrombomodulin, Mesothelin, D2-40, nuclear WT-1, Vimentin in epithelioid cells, CK MNF116 in fibrous cells. Cases where immunohistochemistry indicate an altered diagnosis are filled red. MM=mesothelioma and ADCA=adenocarcinoma. Ext. IHC = extended immunohistochemistry.

| Two-step model (p) | **BerEp** | **CEA** | **CD15** | **Sialyl** | **TTF1** | **EMA** | **Calr** | **HBME** | **Trbm** | **Mesot** | **D2-40** | **WT1** | **Vim** | **MNF** | Primary diagnosis | Ext. IHC |
| --- | --- | --- | --- | --- | --- | --- | --- | --- | --- | --- | --- | --- | --- | --- | --- | --- |
| 0,05 | + | - | - | + | - | - | - | + | - | + | + | + | + | + | MM | ADCA |
| 0,75 | + | - | - | - | - | + | + | + | + | + | + | + | + | + | ADCA | MM |
| 0,03 | - | - | - | - | - | + | + | + | + | + | + | + | + | + | MM | MM |
| 0,01 | - | - | - | - | - | + | + | + | + | + | + | + | - | + | MM | MM |
| 0,11 | - | - | - | - | - | - | - | - | - | - | + | + | - | + | MM | MM |
| 0,00 | - | - | - | - | - | + | + | + | - | + | + | + | + | + | MM | MM |
| 1,00 | - | - | - | - | - | + | + | + | + | + | + | + | + | 0 | ADCA | MM |
| 0,00 | - | - | - | - | - | + | + | + | - | + | + | + | + | + | MM | MM |
| 0,00 | + | - | - | - | - | + | + | + | + | + | + | - | + | 0 | MM | MM |

***Figure S1***

Retest reliability in 19 patients that had been sampled twice at different time points. Regression analysis could not be performed for syndecan-2 since too many values were 0.

***
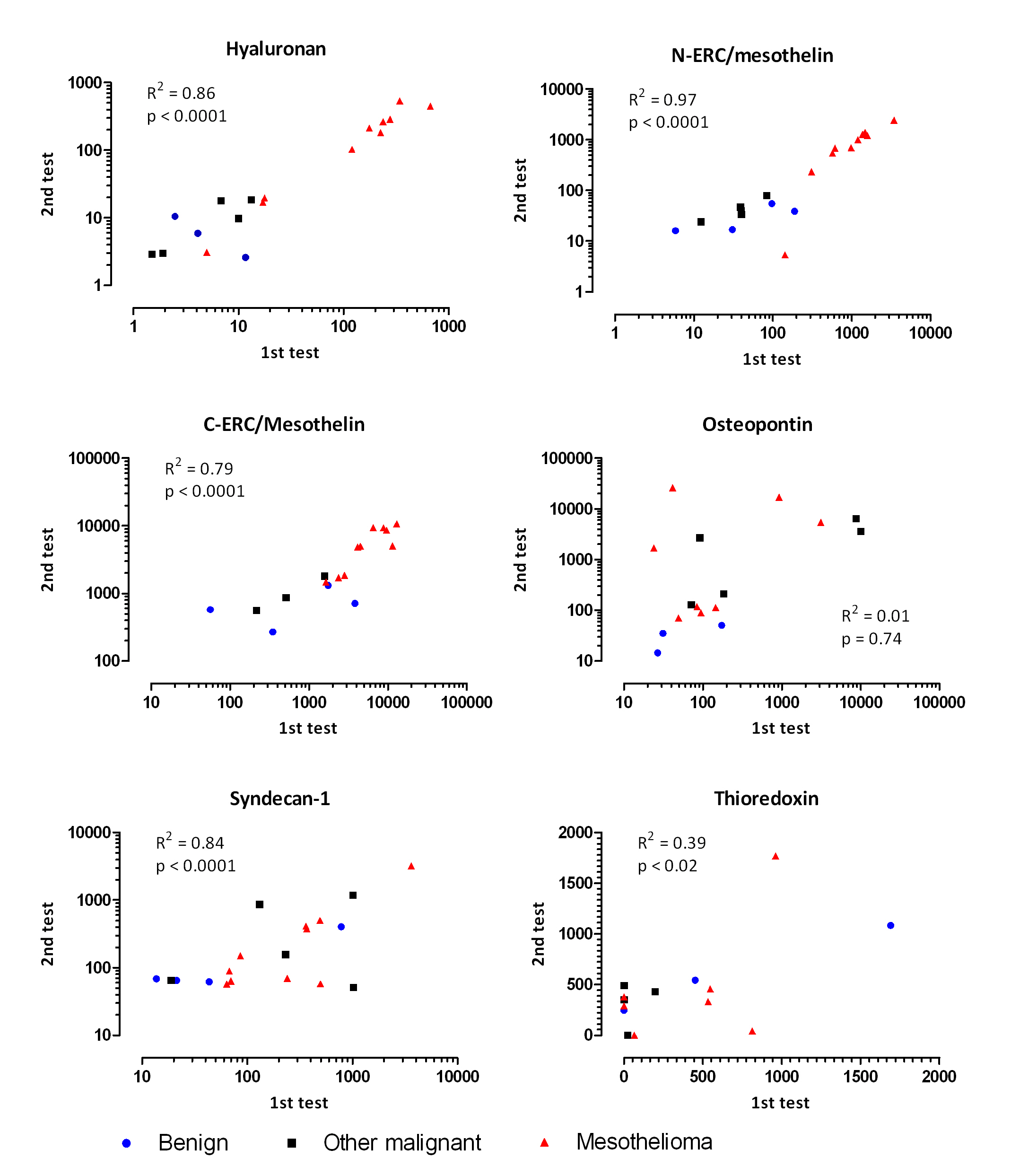
***

***Figure S2***

Linear regression on hyaluronan and N-ERC/mesothelin.


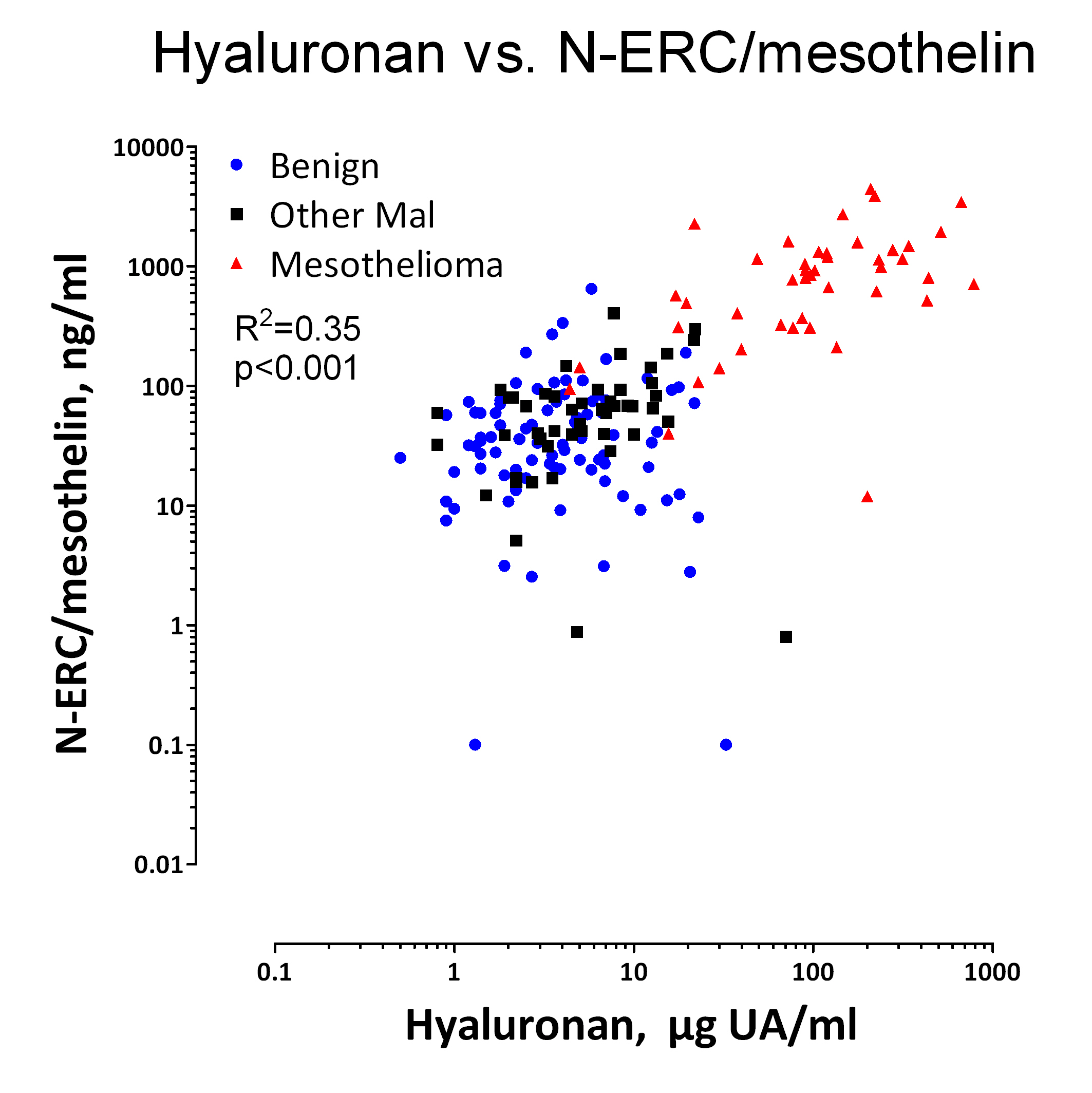

Supplement: File S1 — Includes table S1 and S2 as well as figure S1 and S2. (DOC) [file pone.0072030.s001.doc]
