# Supplementary material for: Hyaluronan and N-ERC/Mesothelin as Key Biomarkers in a Specific Two-Step Model to Predict Pleural Malignant Mesothelioma
Source: PLoS One. 2013 Aug 21;8(8):e72030. doi: 10.1371/journal.pone.0072030 (PMC3749097; doi:10.1371/journal.pone.0072030)
Supplement: File S2 — Appendix with practical examples of how to use the two-step model. (DOCX) [file pone.0072030.s002.docx]

Mundt et al: A biomarker panel predicting mesothelioma

***APPENDIX: EXAMPLES OF APPLICATION OF THE TWO-STEP MODEL IN A CLINICAL CONTEXT***

**Case 1**

A pleural effusion suspected of mesothelioma is found to have a hyaluronan content of 140 µg UA/ml and an N-ERC/mesothelin concentration of 900 ng/ml. The first step in application of the model is to check whether either of the values is above the thresholds; 120 µg UA/ml for hyaluronan, and 1050 ng/ml for N-ERC/mesothelin.

This effusion has a hyaluronan level exceeding the threshold. It therefore supports the diagnosis of a malignant mesothelioma, as the model has a high specificity.

NB. Measuring hyaluronan level is enough in Case 1 to support the diagnosis.

**Case 2**

A pleural effusion from a patient suspected of having mesothelioma is found to have a hyaluronan content of 100 µg UA/ml and an N-ERC/mesothelin concentration of 900 ng/ml. Threshold values are; 120 µg UA/ml for hyaluronan, and 1050 ng/ml for N-ERC/mesothelin.

In this case, neither hyaluronan nor mesothelin exceed their respective thresholds. Therefore, they are entered into the logistic regression model, according to the following formula:

(eq. 1) Predicted risk = e^(b0+ b1*x1 + b2*x2)/(1+ e^(b0 + b1*x1 + b2*x2))

where

b0 = -12.42

b1 = 4.71

x1 = log[Hyaluronan] = log(100)

b2 = 2.71

x2 = log[N-ERC/mesothelin] = log(900)

By entering these values into the equation, we obtain

(eq. 2) Predicted risk = e^(-12.42 + 4.71*log(100) + 2.71*log(900))/(1+ e^(-12.42 + 4.71*log(100) + 2.71*log(900))) = 0.99

The predicted risk is above 0.9, and the values therefore strongly support a diagnosis of a malignant mesothelioma, as the model has high specificity.

**Case 3**

A pleural effusion suspected of mesothelioma is found to have a hyaluronan content of 10 µg UA/ml and an N-ERC/mesothelin concentration of 90 ng/ml.

Threshold values are; 120 µg UA/ml for hyaluronan, and 1050 ng/ml for N-ERC/mesothelin.

Neither hyaluronan nor mesothelin exceed their respective thresholds. They are therefore entered into the logistic regression model, in the same manner as for case 2:

(eq. 3) Predicted risk = e^(-12.42 + 4.71*log(10) + 2.71*log(90))/(1+ e^(-12.42 + 4.71*log(10) + 2.71*log(90))) = 0.08

The predicted risk is below 0.9, and the values do not confirm the diagnosis of mesothelioma. The model, however, has moderate sensitivity and this finding thus does not rule out a mesothelioma.
